# CT and MR Imaging Findings in Methanol Intoxication Manifesting with BI Lateral Severe Basal Ganglia and Cerebral Involvement

**DOI:** 10.5334/jbsr.2836

**Published:** 2022-07-06

**Authors:** Eyup Camurcuoglu, Ahmet Mesrur Halefoglu

**Affiliations:** 1 Sisli Hamidiye Etfal Training and Research Hospital, TR

**Keywords:** diffusion-weighted imaging, magnetic resonance imaging, metabolic acidosis, methanol intoxication, putaminal necrosis

## Abstract

Our case report describes a 54-year-old man who was admitted to our hospital complaining of visual impairment with gastrointestinal and neurological symptoms. Initial computed tomography examination showed bilateral symmetric putaminal and cerebral white matter hypodensities. Evaluation of the following magnetic resonance imaging, restricted diffusion in these corresponding areas were found to be compatible with cytotoxic edema.

**Teaching Point:** Diffusion-weighted imaging (DWI) plays a crucial role in the diagnosis of acute methanol intoxication.

## Introduction

Methanol is a clear, colourless, and scentless liquid, and its acute intoxication usually arises from oral ingestion. It can be absorbed by different body systems including the skin, gastrointestinal tract, and respiratory tract [1].

Although methanol itself is a relatively low toxic agent, following its uptake by the human body, the transformation to toxic metabolites, namely formic acid and formaldehyde, devastating effects occur [2].

## Case Report

A 54-year-old man was admitted to our emergency department complaining of nausea, vomiting, abdominal pain, sudden loss of vision, and deterioration of consciousness. A substantial amount of unknown origin alcohol ingestion was reported by his family during the past day. His initial Glasgow coma scale (GCS) score was 3/15.

Laboratory examination showed elevated liver enzyme levels, (ALT: 56 U/L (<41), AST: 53 U/L (<40), GGT: 116 U/L (10–71)), serum creatinine: 1.92 mg/dl (0.70–1.20), hyperkalaemia: 6.33 mmol/L (3.5–5.3) and hyperglycemia: 353 mg/dl (74–109). Arterial blood gas analysis was performed and revealed severe metabolic acidosis. (PH: 6.87, pO2: 86 mmHg, pCO2: 66 mmHg, HCO3: 5.4 mmol/L, BE: –27 mmol/L).

**Figure 1:**
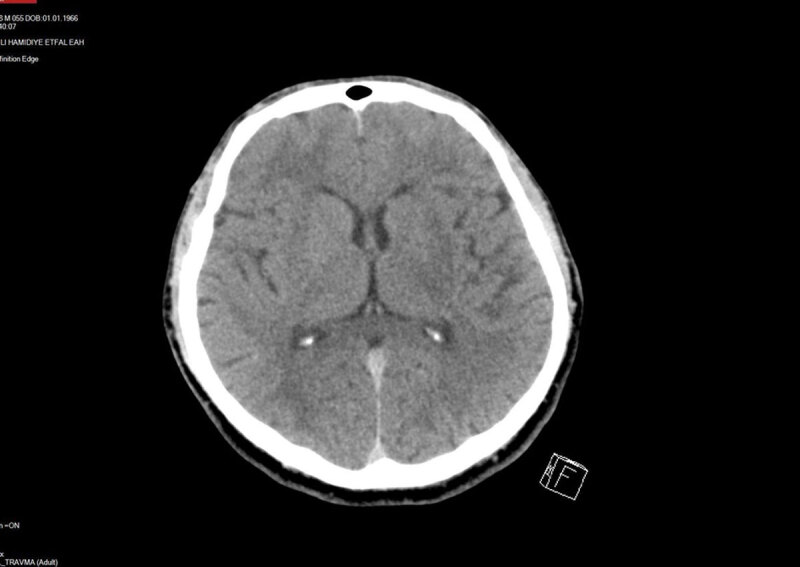
Unenhanced axial CT scan shows bilateral symmetric hypodensities along both lentiform nuclei and cerebral cortical white matter.

**Figure 2:**
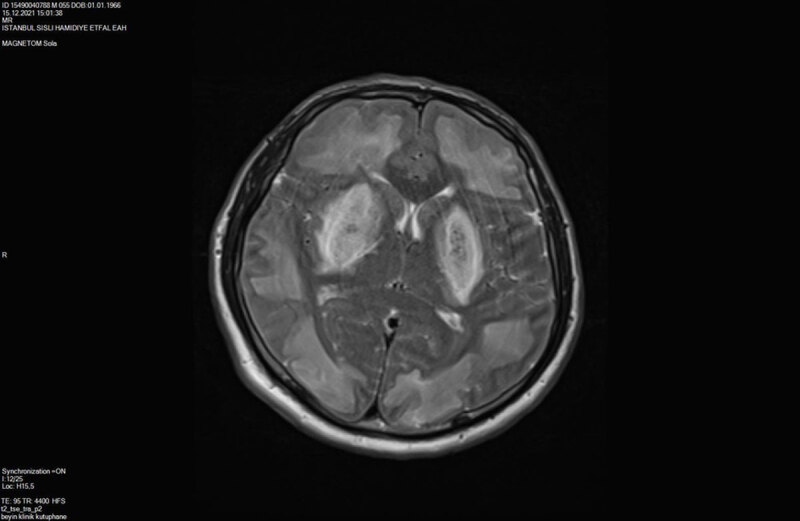
FSE T2-weighted image, bilateral symmetric extensive signal intensity increase along both cerebral hemispheres subcortical region and also bilateral putamina.

**Figure 3:**
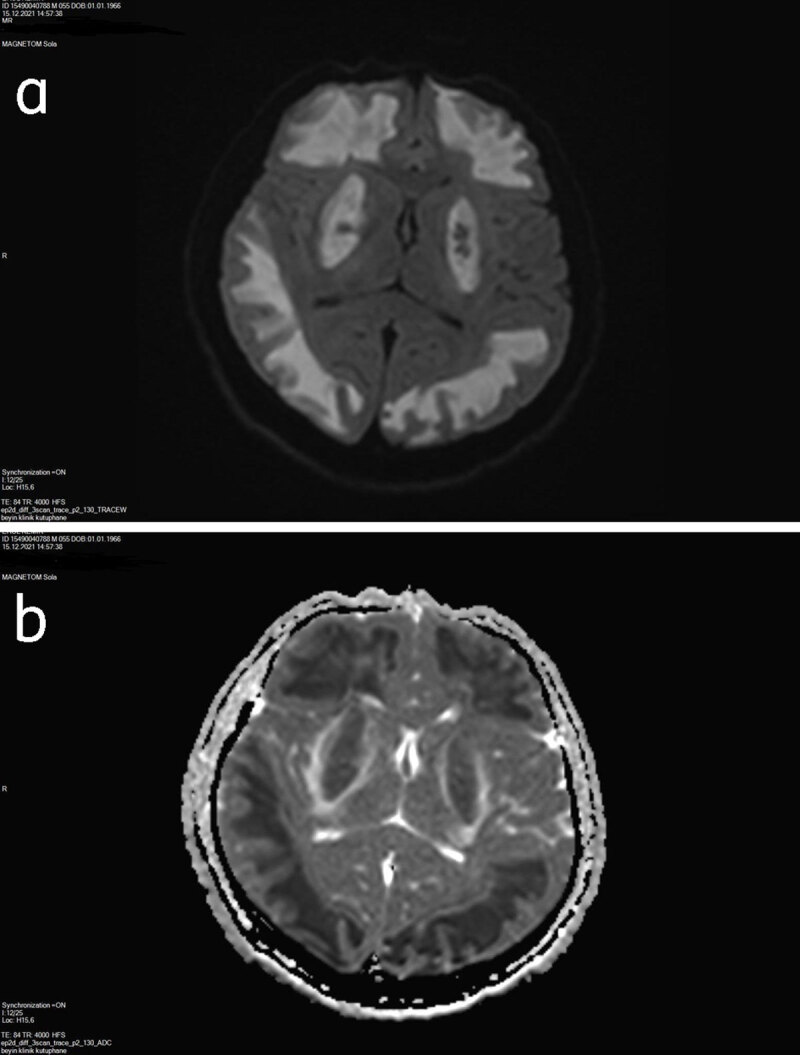
**a** DWI reveals increased signal intensity on both putaminal and cerebral subcortical white matter regions. **b** ADC map shows decreased signal intensity on corresponding brain regions indicating restricted diffusion due to cytotoxic edema.

**Figure 4:**
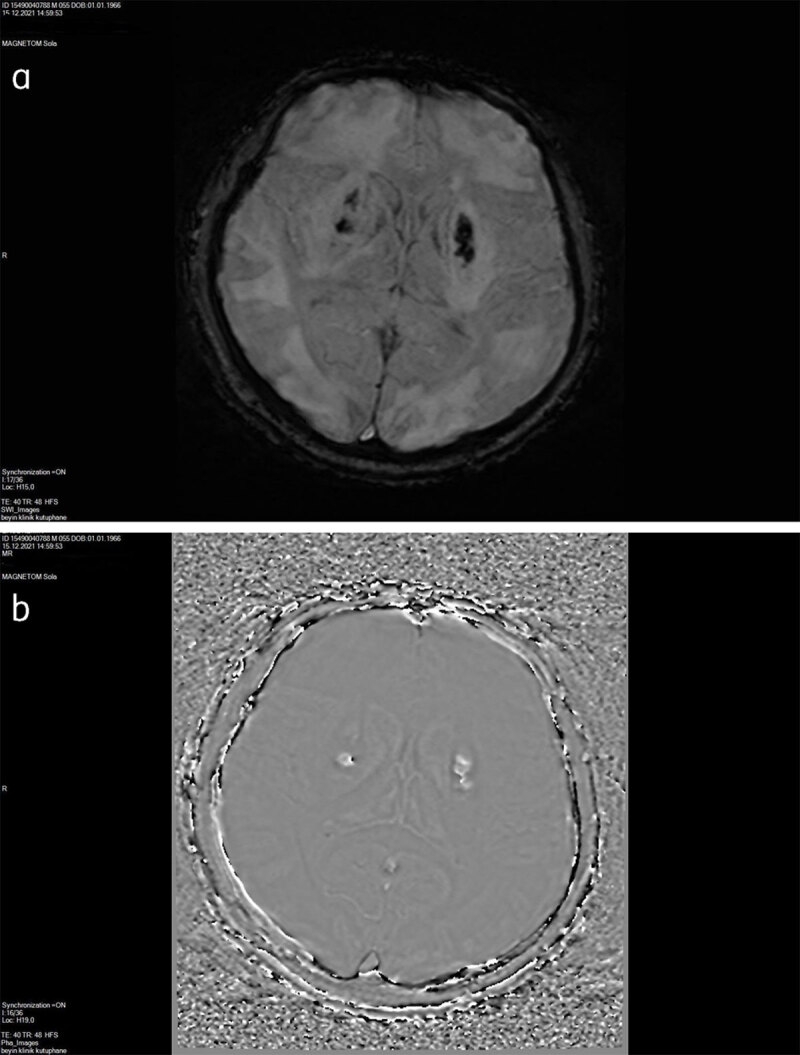
**a** Magnitude SWI demonstrated hypointense hemorrhagic foci on both putaminal regions. **b** Phase-contrast SWI reveals opposite signal intensity due to positive shift effect of deoxyhemoglobin on left-handed MRI system.

An initial unenhanced computed tomography (CT) scan showed bilateral symmetrical hypodensities along both lentiform nuclei and was accompanied by bilateral extensive hypodense subcortical white matter changes (Figure 1). On the third day, brain magnetic resonange imaging (MRI) was performed and on SE T1-weighted and FSES T2-weighted images, bilateral extensive subcortical white matter hypointensity and hyperintensity changes, respectively, along both cerebral hemispheres were detected. Bilateral hyperintense symmetric signal intensity changes were also present along both lentiform nuclei (Figure 2). Diffusion-weighted imaging (DWI) revealed high signal intensity and ADC showed low signal intensity in the corresponding brain regions consistent with restricted diffusion (Figure 3a and b). SWI detected hemorrhagic foci in both lentiform nuclei with a hypointense signal on magnitude images and hyperintense signal on phase-contrast images due to the positive shift effect of paramagnetic deoxyhemoglobin (Figure 4a and b).

The patient was transferred to the intensive care unit, where he was immediately intubated and received intravenous (IV) ethanol; sodium bicarbonate infusion and hemodialysis was performed in order to correct severe metabolic acidosis.

## Discussion

In acute methanol poisoning, following a latent period of 12 to 24 hours, methanol is converted to its toxic metabolites of formic acid and formaldehyde, partly depending on the amount of the ingested dose. These metabolites cause severe toxic effects on the central nervous system (CNS), especially on the basal ganglia and optic nerves, leading to severe metabolic acidosis [3].

In the literature, there are limited number of case reports describing DWI findings of acute methanol intoxication. Deniz et al. [4] reported DWI findings of a methanol intoxication patient and showed bilateral symmetric hyperintensities in the putamen. Similarly, Ahsan [1] and Server et al. [5] demonstrated bilateral putaminal and subcortical white matter lesions with high signal intensity on DWI. Peters et al. [6] showed symmetric hypodensities in the basal ganglia on CT images and marked striatal hyperintensities on DWI, with enhancement after gadolinium injection in an acute methanol poisoning patient. Grasso et al. [7] reported CT and DWI findings of a methanol intoxication case and described a lentiform fork sign indicating development of vasogenic edema in the bilateral external capsules, resembling a fork that borderlines putaminal necrotic regions.

Chen [8] reported an acute methanol intoxication case of a 51-year-old man who had an accidental adulterated spirit ingestion history. Atypical imaging findings were found with rapidly progressing severe subarachnoid hemorrhage and diffuse cerebral edema.

In treatment, IV ethanol administration inhibits methanol dehidrogenation, because both substances use alcohol dehidrogenase enzyme for oxidation. HCO3 replacement is essential to correct metabolic acidosis and stabilizing the patient’s vital parameters carries crucial importance [6].

## Conclusion

MRI including DWI and SWI has an important role in terms of revealing restricted diffusion and hemorrhagic foci, respectively, in the typical brain regions involved.
